# The Variation in the Rhizosphere Microbiome of Cotton with Soil Type, Genotype and Developmental Stage

**DOI:** 10.1038/s41598-017-04213-7

**Published:** 2017-06-21

**Authors:** Qinghua Qiao, Furong Wang, Jingxia Zhang, Yu Chen, Chuanyun Zhang, Guodong Liu, Hui Zhang, Changle Ma, Jun Zhang

**Affiliations:** 1 Key Laboratory of Cotton Breeding and Cultivation in Huang-Huai-Hai Plain, Ministry of Agriculture, Cotton Research Center of Shandong Academy of Agricultural Sciences, Jinan, 250100 P. R. China; 2 Key Laboratory of Plant Stress Research, College of Life Sciences, Shandong Normal University, Jinan, 250014 P. R. China

## Abstract

Plant roots and soil microorganisms interact with each other mainly in the rhizosphere. Changes in the community structure of the rhizosphere microbiome are influenced by many factors. In this study, we determined the community structure of rhizosphere bacteria in cotton, and studied the variation of rhizosphere bacterial community structure in different soil types and developmental stages using TM-1, an upland cotton cultivar (*Gossypium hirsutum* L.) and Hai 7124, a sea island cotton cultivar (*G. barbadense* L.) by high-throughput sequencing technology. Six bacterial phyla were found dominantly in cotton rhizosphere bacterial community including Acidobacteria, Actinobacteria, Bacteroidetes, Planctomycetes, Proteobacteria, and Verrucomicrobia. The abundance of Acidobacteria, Cyanobacteria, Firmicutes, Planctomycetes and Proteobacteria were largely influenced by cotton root. Bacterial α-diversity in rhizosphere was lower than that of bulk soil in nutrient-rich soil, but higher in cotton continuous cropping field soil. The β-diversity in nutrient-rich soil was greater than that in continuous cropping field soil. The community structure of the rhizosphere bacteria varied significantly during different developmental stages. Our results provided insights into the dynamics of cotton rhizosphere bacterial community and would facilitate to improve cotton growth and development through adjusting soil bacterial community structure artificially.

## Introduction

The rhizosphere has been defined as a small area surrounding and influenced by the root^1–3^. Within the rhizosphere, large numbers of microorganisms interact with the plants^4^. Recently, in-depth research has led to an increased understanding of the nature of the rhizosphere. The rhizosphere serves as an important interface for plants-soil-microorganism interaction and signaling, as well as allows an exchange of energy and substances. The rhizosphere microbiome affects plant growth, development, biotic and abiotic stress resistance through altering the absorption of nutrients into plant cells, the exchange of chemical signals, and affects enzyme activity during metabolic processes^5–8^. For example, plant growth-promoting rhizobacteria (PGPR) directly or indirectly provide nitrogen and phosphorus to plants^9–13^, promoting plant growth and development^14, 15^, preventing pathogen colonization and adjusting the resistance of plants to biotic and abiotic stresses^12, 16–19^. Similarly, the plants can regulate the rhizosphere through adjusting the input of material, energy and signals in the soil, thereby changing the community structure of the rhizosphere microbiome^20^. Studies have shown that at least 21% of carbon fixed through photosynthesis of plants has entered the soils by various means, such as root secretions, thus affecting the structure of the microbial community in the rhizosphere^21^. Photoassimilates of cereals transferred to underground reached 30–60%, of which 40–90% were released in the form of root exudates which played a crucial role in the interaction between plant roots and soil microorganisms^22^.

The community composition of the rhizosphere microbiome is affected by many factors, such as ambient conditions, physical and chemical properties of soil, background microbial composition of soil, the stage of plant development and plant genotype^23–27^. Marques *et al*. have shown that both the growth stage and genotype of sweet potato affected the structure of the microbial community in the rhizosphere^27^. Smalla *et al*. compared the bacterial rhizosphere communities in different development stages of strawberry (*Fragaria ananassa* Duch.), oilseed rape (*Brassica napus* L.), and potato (*Solanum tuberosum* L.) grown in the field found that composition of the community structure of the rhizosphere microbiome varied with variations in host plant and developmental stage^28^. The characteristics of the rhizosphere microbiome have previously been reported for some crops, such as rice^29^, corn^30, 31^, wheat^32^, and sweet potato^27^.

Cotton fiber serves as an important natural textile fiber and cottonseed is an important source of forage, food, and oils. Appropriate adjustments of the physiological cycles of cotton plants can improve the plant’s resistance to soil-borne pathogens or suppress colonization of soil-borne disease, decrease the incidence of disease, avoid crop losses arising from adverse environmental factors and cotton production can be improved. There are only few reports on the rhizosphere soil of cottons. The actinobacterial communities in the rhizosphere of transgenic cotton and its non-transgenic parent were analyzed by real-time PCR and denaturing gradient gel electrophoresis, but significant difference was not found^33^. By applying similar approaches, it was shown that cotton rhizosphere plant–microbial interactions are variable in field and significantly influenced by cultivar type^34^. However, until now, no detailed report has been published to defining the rhizosphere bacteria community structure of cotton by high-throughput sequencing technology. The present study analyzed the rhizosphere bacteria of two cotton allotetraploid cultivars under different soil conditions and during different developmental stages. The aims were: (1) to explore the dominant rhizosphere bacterial taxa in cotton rhizosphere and the variations of cotton rhizosphere microbial community in various influence factors; and (2) to identify the specific bacterial communities during different developmental stages. Our study systematically analyzed the change and characteristics in the cotton rhizosphere bacteria during different developmental stages.

## Results

In order to explore the core microbiome of the cotton rhizosphere and its response to soil type, plant genotype and developmental stages, we analyzed the rhizosphere microbiome of two cotton allotetraploid cultivars, upland cotton and sea island cotton, under nutrient-rich soil and cotton continuous cropping field soil during seedling, budding, and flowering stages. The V4 region of the 16 S rRNA gene in bacterial DNA was amplified using the polymerase chain reaction (PCR) technique and sequenced by the Illumina MiSeq platform. A total of 11,883,254 high-quality reads were obtained and combined to 5,907,150 tags, 109,391 tags per sample on average (range: 68,841–138,615) (Supplementary Table S1). The tags were clustered into 13,600 microbial operational taxonomic units (OTUs) at 97% similarity after OTUs that were unassigned and not assigned to the target species were removed (Supplementary Table S1).

### Dominant bacterial phyla of Cotton Rhizosphere Bacterial Community

**Figure 1:**
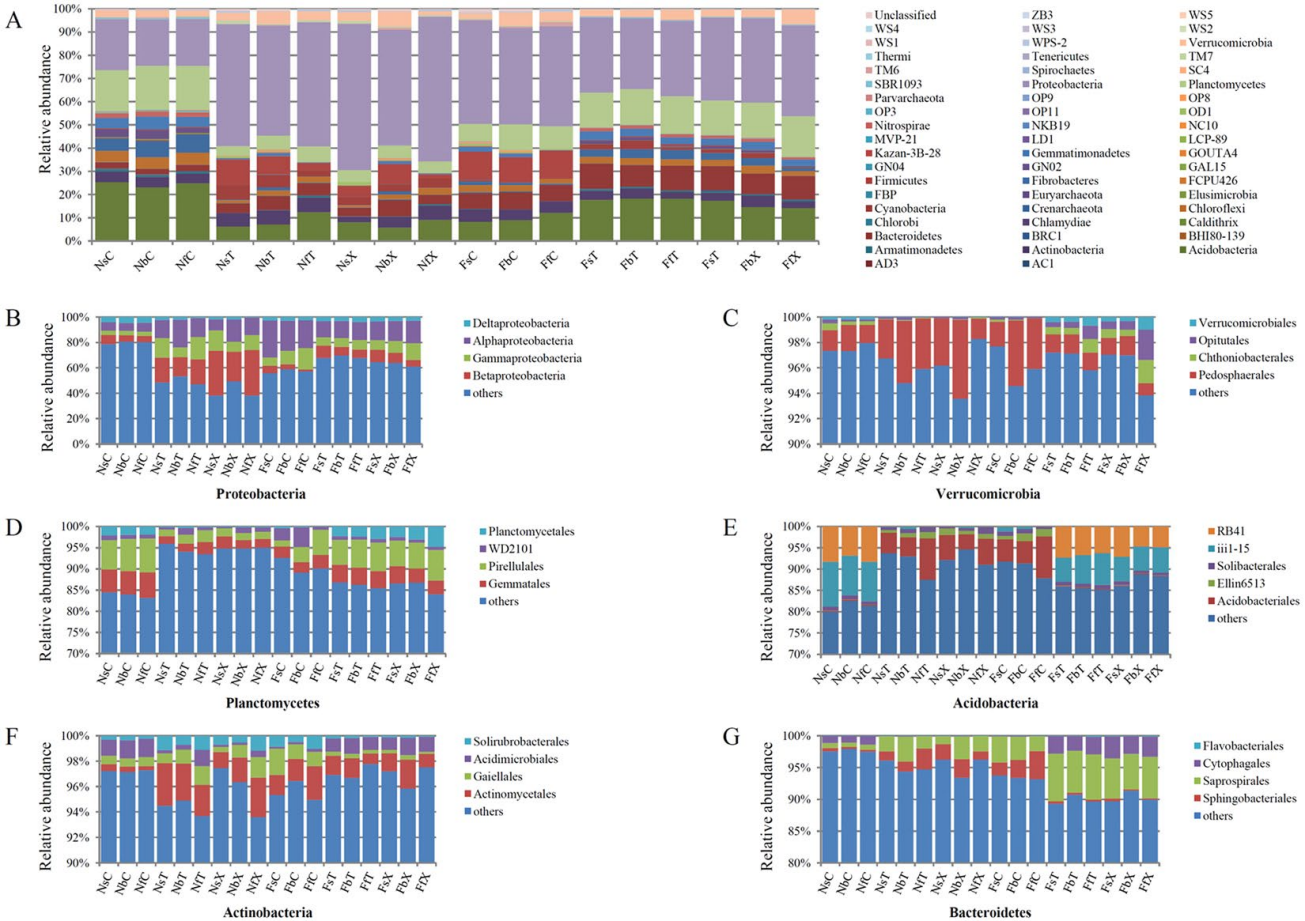
Bacterial community composition in samples of 18 different treatments. Two types of soils were used: nutrient-rich soil (N) and continuous cropping field soil (**F**). Three developmental stages: seedling (s), budding (**B**), and flowering (**F**). Two cultivars: upland cotton (T) and sea island cotton (X), without cotton control (**C**). Each samples was labelled by a three-letter code, such as NsT indicates that seedling of sea island cotton grown in nutrient-rich soil. (**A**) Bacterial community composition by different treatments; (**B**) Proteobacteria, (**C**) Verrucomicrobia, (**D**) Planctomycetes, (**E)** Acidobacteria, (**F**) Actinobacteria, and (**G**) Bacteroidetes community composition in each sample. X-axis shown relative abundance, y-axis are different treatment.

Although the abundance of each phylum varied in cotton rhizosphere soil samples with different treatment, Acidobacteria, Actinobacteria, Bacteroidetes, Planctomycetes, Proteobacteria, and Verrucomicrobia were the six phyla dominant in the cotton rhizosphere of three different development stages and two different soil types, accounting for 75.92–90.17% of all bacterial taxa. Proteobacteria were much more abundant than other phyla, accounting for 30.44–63.00% of the total bacterial taxa in different rhizosphere samples (Fig. 1A; Supplementary Table S2; Supplementary Figures 1 and 2). Some phyla had the same dominant classes or orders in each rhizosphere sample. For example, in the Proteobacteria, the Alphaproteobacteria, Betaproteobacteria, and Gammaproteobacteria dominated in each sample and accounted for 26.98–61.37% of total Proteobacterial microbes (Fig. 1B; Supplementary Table S2). In the Verrucomicrobia, the Pedosphaerales dominated in each sample and accounted for 0.96–6.22% (93.55–98.27% of the Verrucomicrobia were unclassified) of total Verrucomicrobial microbes (Fig. 1C; Supplementary Table S2). The dominant microbes in the Planctomycetes were Gemmatales and Pirellulales in each sample (Fig. 1D; Supplementary Table S2). However, some bacterial phyla had distinct classes and orders in different soils. For example, the dominant microbe of Acidobacteria (except those unclassified) in nutrient-rich soil was Acidobacteriales, accounting for 3.58–9.72% of total Acidobacteria; however, RB41 and iii1–15 were the dominant orders in continuous cropping field soil, accounting for 10.36–13.72% of total Acidobacterial microbes (Fig. 1E; Supplementary Table S2). The dominant microbes of Actinobacteria in nutrient-rich soil were Actinomycetales and Solirubrobacterales; however, Actinomycetales and Acidimicrobiales dominated in continuous cropping field soil (Fig. 1F; Supplementary Table S2). The dominant orders of Bacteroidetes in nutrient-rich soil were Sphingobacteriales and Saprospirales; however, Saprospirales and Cytophagales dominated in continuous cropping field soil (Fig. 1G; Supplementary Table S2).

Dominate phyla in rhizosphere soil were dominantly in bulk soil as well. Besides this, Chloroflexi, Crenarchaeota, Gemmatimonadetes, Euryarchaeota had a higher relative abundance in nutrient-rich soil, and Firmicutes, Chloroflexi had a higher relative abundance in field soil (Fig. 1A; Supplementary Table S3; Supplementary Figures 1 and 2). Different bacterial phyla experienced varying degrees of effects from cotton roots. For example, in nutrient-rich soil, Proteobacterial abundance in rhizospheric soil was 2.65 +/− 0.25 times higher than that of bulk soil, and the abundance of Firmicutes in rhizospheric soil was 17.09 +/− 8.65 times of that in bulk soil highly than that of Proteobacteria, but the abundance of Acidobacteria in the rhizospheric soil was lower than bulk soil for about 0.33 +/− 0.08 times (Supplementary Table S4).

### Variation of cotton rhizosphere bacterial community in different soils

We analyzed the difference of bacterial relative abundance in rhizosphere soil compare with bulk soil between different soil types. There were different effects of cotton root on the same bacterial community between different soil types. For example, in nutrient-rich soil the relative abundance of Proteobacteria in rhizospheric soil was 2.61 +/− 0.27 times higher than that in bulk soil. However, in continuous cropping field soil, relative abundance of Proteobacteria in rhizospheric soil was 0.80 +/− 0.08 times of that in bulk soil. (Supplementary Tables S2 and S4). Besides this, the number of OTUs of bacteria was changed differently between two soil types. Rhizospheric microbial OTU number (1026–2609) in nutrient-rich soil was lower than that in bulk soil (5096–5152). However, in continuous cropping field soil, the number of OTUs of rhizosphere bacteria (4182–4961) was higher than that in bulk soil (1176–2568; Supplementary Table S5).

Bacteria communities, the relative abundance in rhizosphere soil increased or decreased compare with bulk soil, were different between two types of soils. Some bacterial genus had initially exhibited very low relative abundance even barely detectable were greatly increased in the rhizosphere, while some genus with high relative abundance were greatly decreased in the rhizosphere (Supplementary Tables S6). For example, in nutrient-rich soil, the abundance of Burkholderia in the rhizosphere soil was significantly higher than that in bulk soil (*P* < 0.01), and the abundance of Candidatus Nitrososphaera in the rhizosphere was significantly lower than that in bulk soil (*P* < 0.01; Table 1; Supplementary Table S6). Bacterial genus, the relative abundance in rhizosphere were lower than that in bulk nutrient-rich soil, such as Candidatus Nitrososphaera, Gemmata, Pirellula, Planctomyces and Streptomyces, were higher than bulk soil from cotton continuous cropping field. However, bacterial genus, the relative abundance in rhizosphere were higher than that in bulk nutrient-rich soil, such as Alkanibacter, Bradyrhizobium, Devosia, Lactococcus, Phenylobacterium, and Rhodoplanes, were lower than bulk soil from cotton continuous cropping field (Table 1; Supplementary Table S6).

**Table 1:** Bacteria that effected opposite by cotton root in two soil types (“−” denotes bacteria that were less abundant in pots containing cotton plants, “+” denotes bacteria that were more abundant in pots containing cotton plants).

Bacteria	Relative abundance in nutrient-rich soil (mean)		Relative abundance in field soil (mean)	
	Rhizosphere	control	rhizosphere	control
*Candidatus_Nitrososphaera*	0.0709−	6.7730	3.1273+	0.2685
*Planctomyces*	0.2081−	2.0322	3.0221+	0.2345
*Pirellula*	0.0418−	1.4558	1.2115+	0.0800
*Gemmata*	0.1629−	1.3748	1.2058+	0.3745
*Steroidobacter*	0.0116−	0.6343	0.2664+	0.0557
*Lactococcus*	5.4577+	0.0014	0.0994−	10.5074
*Rhodoplanes*	2.6595+	0.9710	0.8658−	5.6081
*Devosia*	1.3467+	0.0373	0.3187−	0.8213
*Alkanibacter*	0.1745+	0.0000	0.0001−	0.5717
*Phenylobacterium*	0.5397+	0.0625	0.1767−	0.7362
*Bradyrhizobium*	1.0475+	0.1672	0.696458−	1.440519

### Changes in the cotton rhizosphere bacterial community during different developmental stages

**Figure 2:**
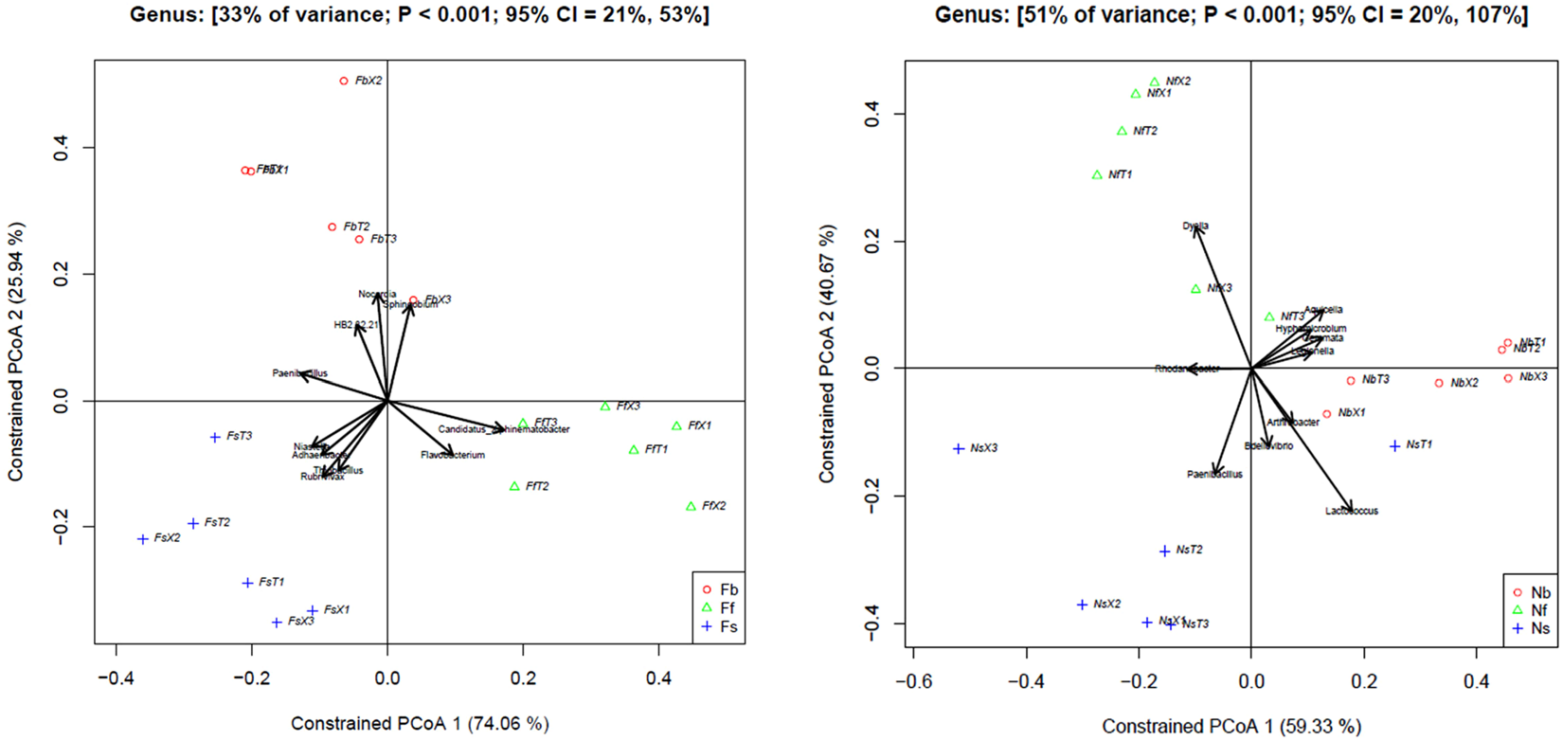
Canonical analysis of principal coordinates analysis (CAP) of influence to rhizosphere bacteria of development stage in two soil types. (**A**) Variation between samples in Bray-Curtis distances constrained by development stage in cotton continuous cropping soil (33% of variance; *p* < 0.001) and (**B**) Variation between samples in Bray-Curtis distances constrained by development stage in nutrient rich soil (51% of variance; *p* < 0.001).

We used a canonical analysis of principal coordinates (CAP) to better quantify the influence of the development stage on the β-diversity. Influence on rhizosphere microbiome was difference in two soil types. Effect of development stage to rhizosphere microbiome in nutrient soil is stronger than that of field soil. Development stage explained 51% of the variance in nutrient-rich soil but 33% in cotton continuous cropping field soil (Fig. 2).

With changes of developmental stages, the total number of OTUs of rhizosphere bacteria in continuous cropping field soil decreased (mean of Upland cotton (T): 4958 (seedling stage), 4848 (budding stage), 4723 (flowering stage); mean of Sea Island cotton (X): 4961 (seedling stage), 4840 (budding stage), 4182 (flowering stage). The similar series of three numbers are used below to indicate the three stages). The total number of OTUs of rhizosphere bacteria in nutrient-rich soil first increased and then decreased with the change of developmental stages (T: 1477, 2609, 1463; X: 1026, 2447, 1513), the highest in the budding stage (Supplementary Figure 3; Supplementary Table S5). In continuous cropping field soil, stage specific OTUs were largest in seedling stage (T: 721; X: 823), whereas in nutrient-rich soil, the budding stage had the largest stage specific OTU number (T: 1263; X: 1796). In addition, the number of OTUs that the three stages shared in common in continuous cropping field soil (T: 4930; X: 4623) was significantly higher than that in nutrient-rich soil (T: 1365; X: 1037) (Supplementary Figure 3; Supplementary Tables S7).

**Figure 3:**
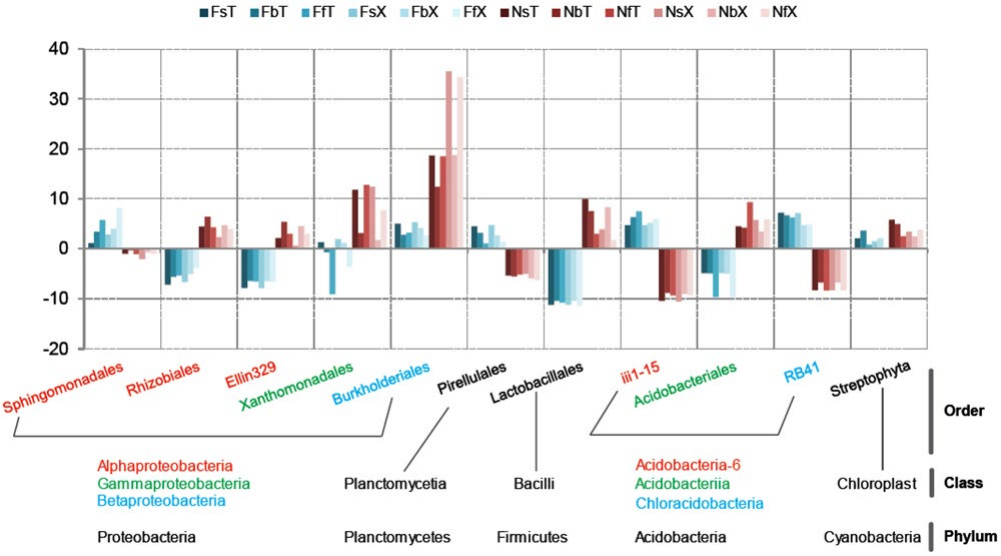
Analysis of increased and reductions of the abundance of major bacteria in two types of soils at different developmental stages.

Significantly difference of bacterial community compositions in the cotton rhizosphere was observed between different development stages (Figs 1,2 and 3; Supplementary Tables S3 and S5). We found microbes exhibited great differences in relative abundance between rhizosphere and bulk soil were mainly distributed in five bacterial phyla, namely Acidobacteria, Cyanobacteria, Firmicutes, Planctomycetes and Proteobacteria by analyzing relative abundance at each stage (Fig. 3). In addition, the bacterial community structure showed large differences in various developmental stages in both continuous cropping field and nutrient-rich soil. Among bacterial communities, which relative abundance in rhizosphere soil were higher than bulk soil in nutrient-rich soil, such as Acidobacteriales, Burkholderiales and Xanthomonadales, had greater increase of relative abundance in seedling and flowering stages. Ellin329 and Rhizobiales had a maximal increase in relative abundance in the budding stage (Fig. 3; Supplementary Table S8). Among bacterial communities, which relative abundance in rhizosphere soil were higher than bulk soil in continuous cropping field soil, the increase in the relative abundance of iii1–15 and Sphingomonadales were smallest in the seedling stage and gradually increased in the budding and flowering stages. In contrast, Burkholderiales, Pirellulales, and RB41 had a maximum increase in the seedling stage. In addition, Streptophyta showed a large abundance increase in the budding stage (Fig. 3; Supplementary Table S8).

Bacterial orders which relative abundance increased in rhizosphere soil compare with bulk soil were analyzed. The abundance of Burkholderiales and Saprospirales during the three developmental stages of two cultivars was found to be higher than that in bulk soil in both soil types, suggesting these taxa were the common core bacteria of the rhizosphere of both cultivars. The relative abundance of Streptophyta and Pseudomonadales in the rhizosphere at three developmental stages of upland cotton was higher than that in bulk soil, indicating they were specific core rhizosphere bacteria of upland cotton TM-1. We further analyzed specific bacterial orders at different developmental stages and found that Xanthomonadales in the seedling stage, Rickettsiales, Opitutales, and Pseudomonadales in the budding stage, and Solibacterales in flowering stages were shared bacterial orders of two cultivars, whose abundance was higher than that in bulk soil (Supplementary Table S9).

Analysis of cotton plant growth-promoting rhizobacteria (PGPR) showed that relative abundance of most nitrogen-uptake-related bacteria genera, such as *Mesorhizobium*, *Sinorhizobium*, *Rhizobium*, and biocontrol genera, such as *Lysobacter* increased significantly (*P* < 0.05; Supplementary Table S10). Additionally, the abundance of nitrogen-fixing genera increased in the budding stage, and biocontrol species showed a higher abundance in the seedling and flowering stages compare with budding stage, but not statistically significant (Supplementary Table S10). The relative abundance of phosphorous bacterium *Arthrobacter* was significantly lower than control (*P* < 0.05).

### α-Diversity Comparison among Different Samples

Five indices, including the Observed Species (Sobs), Chao, Abundance Based Coverage Estimator (ACE), Shannon, and Simpson indices, were used to measure the α-diversity of each sample. Among these indices, the Sobs, Chao, and ACE indices reflect species richness in samples, without considering the abundance of each species. Meanwhile, the Shannon and Simpson indices reflect the species diversity of the community, and are affected by species richness and species evenness of the sample community. The corresponding dilution curve of the first three indices may also reflect whether the sample sequencing depth basically covered all the species in the sample. The dilution curve supported the presence of a sufficient sequencing depth in this experiment (Supplementary Figure 4).

**Figure 4:**
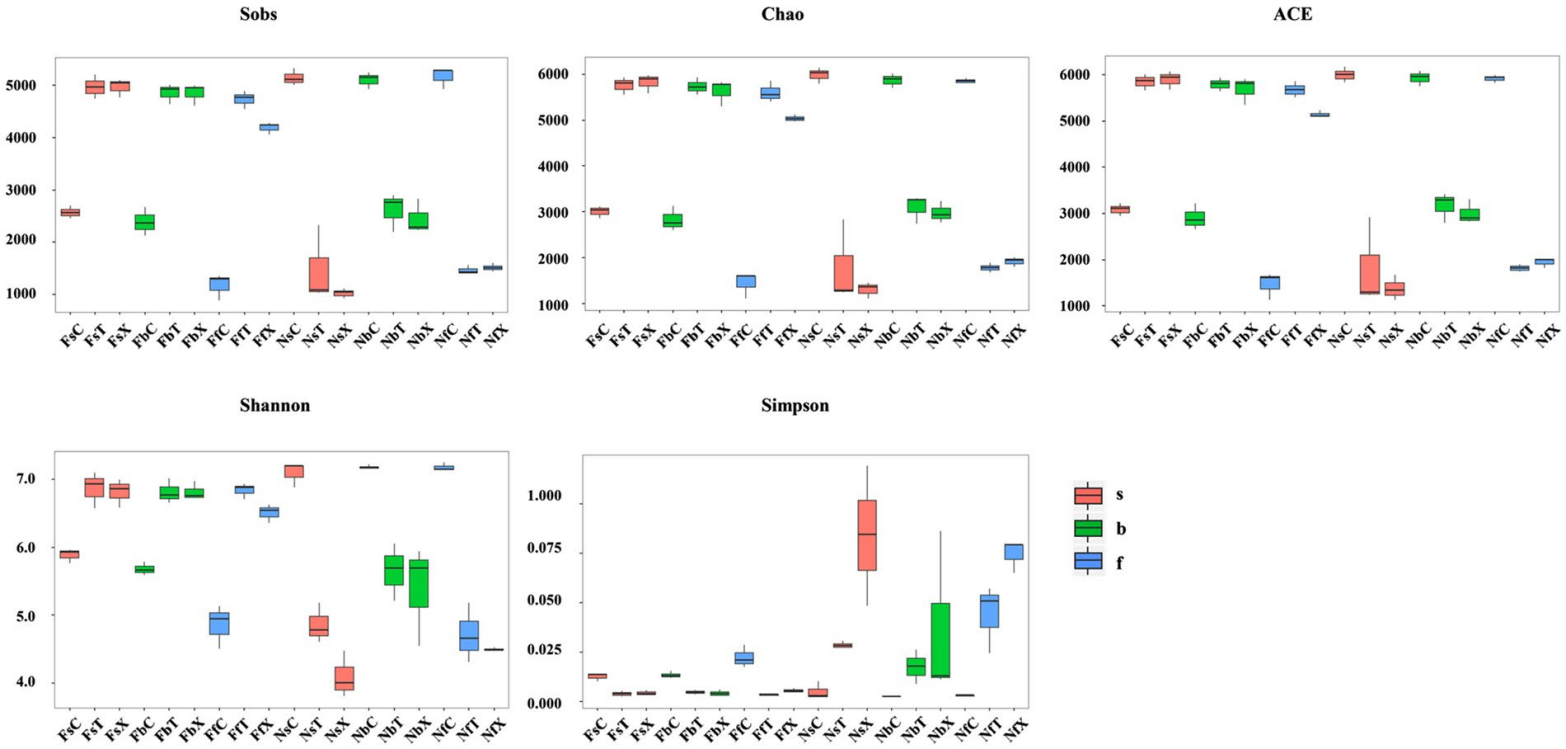
Bacterial α-diversity in each sample. From left to right and from top to bottom, box plots are Sobs, Chao, ACE, Shannon, and Simpson indices.

Changes in α-diversity in nutrient-rich soil were greater compare with continuous cropping field soil (Fig. 4; Supplementary Figure 5; Supplementary Table S11). The α-diversity of rhizobacteria in continuous cropping field soil were increased compared with bulk soil (Sobs, Chao, ACE: *P* < 0.05; Shannon, Simpson: *P* > 0.05), but reduced in nutrient-rich soil (*P* < 0.01). The α-diversity in the bulk soil of nutrient-rich soil was significantly higher than that of continuous cropping field soil (*P* < 0.05). However, rhizobacteria α-diversity of nutrient-rich soil was significantly lower than that of continuous cropping field soil (*P* < 0.01; Fig. 4; Supplementary Table S11).

Besides the difference in different soils, the microbial α-diversity of samples also showed significant differences at different developmental stages. In continuous cropping field soil, the Sobs, Chao, and ACE species indices of rhizobacteria gradually decreased with the change from the seedling to budding to flowering; this was consistent with the results of OTU number in community composition analysis. In the flowering stage of TM-1 (*G. hirsutum*) and budding stage of Hai 7124 (*G. barbadense*), the Shannon and Simpson diversity indices were greater than that of a previous developmental stage (Supplementary Table S11). However, in nutrient-rich soil, changes of two cultivars were the same. α-diversity of the budding stage was significantly higher than that of the other two stages (Supplementary Table S12) consistent with the results of OTU number in community composition analysis. In nutrient-rich soil, α-diversity of TM-1 were significantly (*P* < 0.05) higher than that of Hai7124 in seedling and budding stages, but lower significantly in flowering stage (*P* < 0.05). In continuous cropping field soil, the α-diversity of rhizobacteria of TM-1 was lower than that of the Hai 7124 in seedling stage, but higher in budding and flowering stages (*P* < 0.05; Supplementary Table S12).

### β-Diversity Analysis in Samples

**Figure 5:**
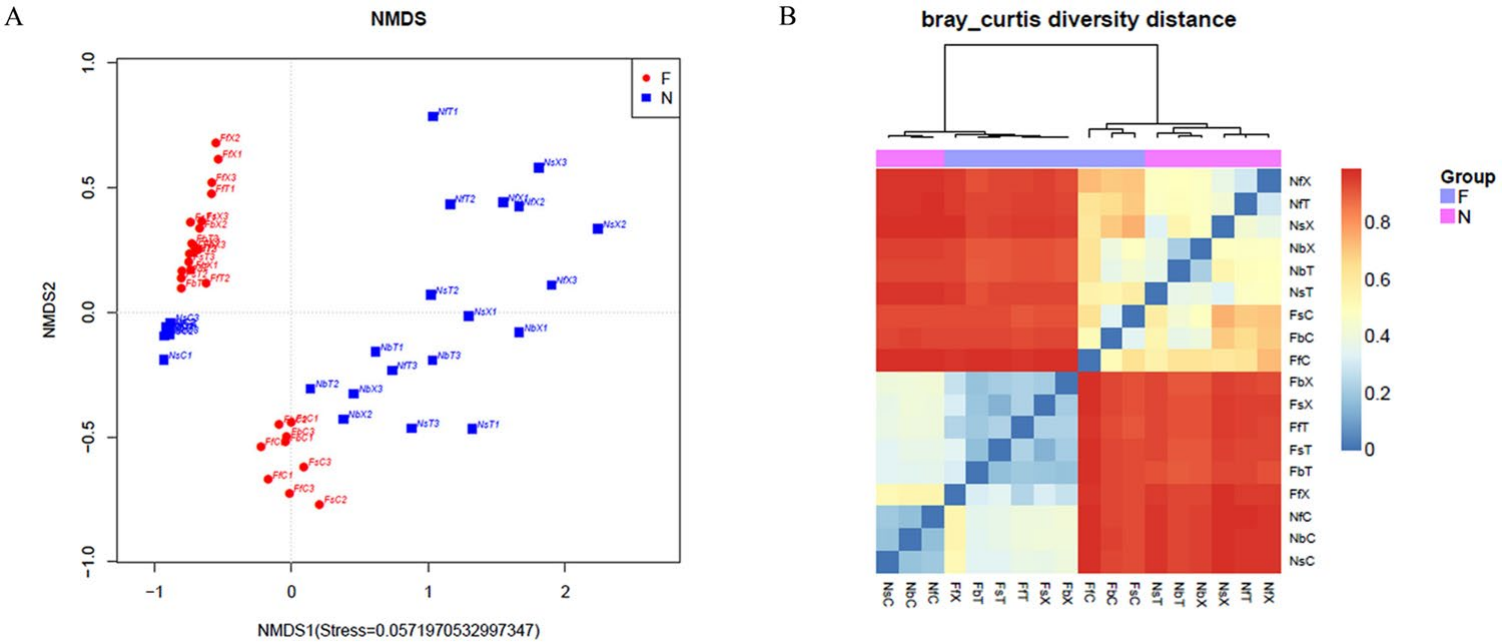
β-diversity analysis of different treatments. (**A**) Cluster analysis of different treatments. (**B**) Bray–Curtis distance analysis of different treatments.

NMDS (Non-Metric Multi-Dimensional Scaling) analysis based on Bray–Curtis showed that rhizosphere and bulk soil samples from the same type of soil clustered to form one community, but the rhizosphere soil and bulk soil have large difference (Fig. 5A). β-diversity analysis also showed the largest difference in different soils (weighted UniFrac, 1.60; unweighted UniFrac, 0.68; Bray–Curtis, 0.93), followed by the difference at different developmental stages (weighted UniFrac, 0.69; unweighted UniFrac, 0.24; Bray–Curtis, 0.39). β-diversity of different genotypes was the smallest (weighted UniFrac, 0.59; unweighted UniFrac, 0.18, Bray–Curtis, 0.32), consistent with the results of NMDS analysis (Fig. 5B; Supplementary Table S13). To assess the effects of different factors on the structure of the bacterial community of the cotton rhizosphere, we used statistical methods to analyze Bray–Curtis diversity at the family level and found that soil factors contributed about 54.03% of the structure of the cotton rhizosphere microbial community, and developmental stages and genotype contributed about 19.23% and 12.39% respectively, interaction of each two factors contributed about 14.35% (Supplementary Table S16).

In addition, CAP analysis indicated that β-diversity in different soil type were difference (Fig. 2). β-diversity of nutrient-rich soil at different developmental stages (weighted UniFrac, 0.71–0.96; unweighted UniFrac, 0.36–0.43; Bray–Curtis, 0.42–0.53) and between different cultivars (0.59–0.80, 0.36–0.43, and 0.42–0.53 for the three indices, respectively) was significantly higher than that of continuous cropping field soil at different developmental stages (*P* < 0.01; 0.45–0.58, 0.20–0.32, and0.34–0.43, respectively) and between different cultivars (0.41–0.51, 0.09–0.10, and 0.23–0.28, respectively). The effect to bacterial community structure of cotton root in nutrient-rich soil was greater than in continuous cropping field soil (Fig. 5B; Supplementary Table S13). This finding was consistent with the α-diversity results.

## Discussion

### Differences of Microbial Community Structure between Cotton Rhizospheric Soil and Bulk Soil

Previous studies have shown that plant roots can affect the community structure of the rhizosphere microbiome by changing the physical and chemical properties of soil. As shown in the present study, Acidobacteria, Actinobacteria, Bacteroidetes, Planctomycetes, Proteobacteria and Verrucomicrobia were more abundant than other microbes in both nutrient-rich soil without previous cotton planting and continuous cotton-cropping field soil as well as the corresponding rhizosphere soil. However, these six bacterial phyla in rhizosphere soil were either promoted or inhibited to different degrees when compared with bulk soil. These data suggest that soil conditions play an important role in determine cotton rhizosphere bacterial community. Lundberg *et al*. found that seven bacterial phyla, namely Proteobacteria, Bacteroidetes, Actinobacteria, Acidobacteria, Firmicutes, Gemmatimonadetes, and Cyanobacteria, dominated in rhizosphere soil of *Arabidopsis* ^23^. Ling *et al*. studied rhizosphere microorganisms in own-root watermelon, own-root bottle, gourd and grafted-root watermelon, and showed that Acidobacteria, Actinobacteria, Bacteroidetes, Cyanobacteria, Firmicutes and Proteobacteria, were the most dominant^35^. Davide *et al*. found that Actinobacteria, Bacteroidetes and Proteobacteria were the dominant rhizosphere bacterial phyla of barley^36^. Although dominant bacterial phyla in rhizosphere is different among different plants, Actinobacteria, Bacteroidetes and Proteobacteria were shared as dominant bacterial phyla of cotton plants and the above plants, indicating that they may be the most common dominant bacterial phyla of plant rhizosphere bacteria. In addition, compared with other plants, Planctomycetes and Verrucomicrobia were very abundant in cotton rhizosphere soil, suggesting that this could be a result of specific root exudates of cotton. This topic awaits further research.

### Soil is a Key Factor Determine the Rhizosphere Microbial Community Structure of Cotton

Zarraonaindia showed that, as a potential microorganism library of plant-associated microorganisms, soil microorganisms have a strong influence on grape root-associated microorganisms^37^. Soil background microorganisms are the main cause of the variation in the rhizosphere microbiome community structure in different types of soils^38^. In this study, a significant difference was observed in cotton rhizosphere bacterial community structure between continuous cropping field soil and nutrient-rich soil. This presumably occurred mainly because of a difference in community structure in these two soils. Besides this, the nutrient-rich soil has a loose texture and its nitrogen-phosphorous-potassium, organic matter and trace elements were significantly higher than that in continuous cropping field soil (Supplementary Table S14). Thus, different physical and chemical environments of the two soils probably resulted in different degrees of promotion or inhibition or reverse effect of the same bacterial community by cotton roots.

Our results demonstrated that cotton roots have different effects on α-diversity of the rhizosphere bacterial community in the two types of soils analyzed here. α-diversity of rhizosphere bacteria inhibited in nutrient-rich soil, but promoted in continuous cropping field soil by cotton roots significantly. In addition, β-diversity of nutrient-rich soil was significantly higher than that of continuous cropping field soil (Fig. 5B; Supplementary Table S13). We speculate that the physical and chemical characteristic of nutrient-rich soil maximizing the reproduction of bacteria, without the interference from external factors. However, plants will change the physical and chemical properties of nutrient-rich soil, and secrete some substances to modulate root microorganisms according to their needs. So, many microorganisms that were originally present in nutrient-rich soil, failed to adapt to the changing environment, experienced a reduction in their abundance or even disappeared. Inversely, a portion of microbes was promoted and multiplied greatly; thus creates a competitive relationship with other microbial species or secrete antimicrobial substances to inhibit the colonization of other bacterial species. Therefore, richness of rhizosphere bacteria in nutrient-rich soil are significantly lower than that in bulk soil. In the other hand, long-term selection of cotton roots on soil bacteria in continuous cropping field soil, a portion of microbes gradually disappear or in a very low level. Thus, the species richness of bulk soil was significantly lower than that in nutrient-rich soil. This is consistent with previous studies that continuous cropping reduced the diversity of the structure and function of the soil microbial community^11, 39, 40^. In continuous cropping field soil planted with cotton, some bacteria, at low abundance or not detectable of OTUs, were promoted by cotton and their abundance increased greatly (Supplementary Table S15; Supplementary Figure 6). Therefore, the species richness of rhizosphere bacteria in continuous cropping field soil was significantly higher than that in bulk soil.

### Variation of Rhizosphere Bacterial Community during Different Developmental Stages of Cotton

Baudoin *et al*. proposed that, as plants develop, the quantity and quality of the root exudates change, leading to variations of the rhizosphere microbial community composition present during different developmental stages^41^. Other studies have also demonstrated that rhizosphere microbes were significantly affected by the developmental stages of plants^30, 42–44^. Our results indicate that the community composition of cotton rhizosphere bacteria varied significantly during different developmental stages. The richness of cotton rhizosphere bacteria peaked in seedling stage in continuous cropping field soil and budding stage in nutrient-rich soil. Promotion or inhibition to bacteria relative abundance was varied during different development stages. In nutrient-rich soil, auxo-action on Rhizobiales and Ellin329 peaked in the budding stage. In continuous cropping field soil, auxo-action on Sphingomonadales and iii1–15 by cotton roots gradually increased with change in the developmental stages. We suggest that the promoted bacteria at each stage may represent the bacterial taxa that are required for the growth of the plant during that developmental stage. This awaits further research.

Rhizosphere microbiome affect plant growth by secreting plant hormones, improving soil nutrient availability and enhancing resistance to pests and diseases, etc. In addition, studies have shown that microbes regulated the flowering stage of some plants (*Boechera stricta*, *Arabidopsis thaliana*, and *Brassica rapa*)^45, 46^. Analysis of the cotton rhizosphere PGPR showed that the nitrogen-uptake-related bacteria, such as the genera *Mesorhizobium*, *Nitrospira, Rhizobium*, and *Sinorhizobium* is increased, and biocontrol genera, such as *Agrobacterium* and *Lysobacter*, had a significant increase in relative abundance compared with the bulk soil. In addition, the abundance of nitrogen-fixing bacteria increased in the budding stage, which may be related to the enhanced nutrient requirements of plants during this stage. The biocontrol bacteria had significantly higher relative abundance in the seedling and flowering stages than in the budding stage, played a protective role for plants during a period of high incidence of soil-borne diseases.

## Conclusions

In this study, we report two different dynamics of bacterial communities in rhizosphere of different soil types, field soil that cotton continuous cropped for many years and nutrient-rich soil without any cropping before, during different development stages. Remarkable differences of rhizosphere bacterial community composition were observed from cotton growing in different soils, at different developmental stages, and of different genotypes. This will help researchers to explore specific microbes and their functional genes that are required for cotton growth under different growth conditions and during different developmental stages. In addition, this study also lays the basis of providing bacterial fertilizer for cotton at the different developmental stages, regulating the development of cotton, and increasing the resistance of cotton plants to soil-borne diseases; especially for bacterial fertilizer that is resistant to cotton *Verticillium* wilt, a disease also known as cotton cancer. This knowledge will be also vital for the development and use of bioorganic fertilizers, reduced use of chemical pesticides and fertilizers, and protection of farmland ecosystems.

## Material and Methods

### General strategy

We investigated the rhizospheric microbiomes of two varieties of cotton across a serious of experimental variables: soil type, plant genotype and plant developmental stage. Soils included continuous cropping field soil and nutrient-rich soil. *Gossypium hirsutum* cv. TM-1 and *G. barbadense* cv. Hai 7124 were selected as two different genotypes studied here. Plant the cotton in continuous cropping field soil and nutrient-rich soil in greenhouse; and make sure the environment conditions were uniformed. The soil samples were collected at seedling, budding and flowering stages respectively.

### Plant materials

Seeds of *Gossypium hirsutum* cv. TM-1 and *G. barbadense* cv. Hai 7124 was provided by professor Zhang Tian-Zhen, Cotton Research Institute of the State Key Laboratory of Crop Genetics and Germplasm Enhancement at Nanjing Agricultural University.

### Soil

Nutrient-rich soil was produced by Feng Yuan Science and Technology Ltd. Field soil was sampled from a continuous cropping cotton field at the Lin Qing Experiment Station of the Shandong Cotton Research Center. Samples were obtained from 15 to 30 cm below the surface soil layer for our experiment. Visible weeds, twigs, worms, and insects were removed, then the soil was crushed with an aluminum mallet to a fine consistency and sifted through a sterile 2-mm sieve. Because the sieved soil was poorly drained and sampling the rhizospheric soil proved to be difficult, we adopted the practice of mixing sterile sand into soils at a soil: sand ratio of 2:1 following Lundberg *et al*.

### Soil analyses

Soil analysis included measures of available N, P, K, Cu, Zn, Fe, Mn as well as pH, organic matter, exchangeable Ca, Mg, Na in field soil, a soil sand mixed sample and nutrient-rich soil were analyzed by Key Laboratory of Plant Nutrition and Fertilizer of Shandong Province.

### Plant germination, transplant, and cultivation in the greenhouse

Seeds of *G. hirsutum* cv. TM-1 and *G. barbadense* cv. Hai 7124 were delinted, surface sterilized for 15 min in 75% ethanol followed by 30 min in 30% H_2_O_2_, then rinsed five times with sterile distilled water. Seeds were germinated on 1% water agar overlaid with sterile paper and incubated at 28 °C in the dark until roots were 2–3 cm long (2–3 d). After germination, the cotton seedlings were transplanted into the various soils and seedling were raised in a tissue culture room at 28 °C. Twenty-seven pots were prepared for each kind of soil, nine of TM-1, nine of Hai 7124 and nine controls. Plants were moved to a greenhouse as soon as the seedlings developed a second real leaf. The pots were watered every three days with sterile water. Pots were watered with 500 ml per pot in the seedling stage and 1000 ml per pot in budding and flowering stages. All weeds were manually removed from the pots when identified.

### Sampling of the rhizosphere and bulk soil

Each pot was inverted to remove the soil and plant, and the plant was gently shaken to remove the soil that did not adhere to the root surface. Rhizospheric soil included ~1 mm of soil that tightly adhered to the root surface and was not easily shaken from the root. To separate the soil that adhered to the roots directly, the roots with attached soil were placed in a sterile flask with 50 ml of sterile buffered phosphate saline solution and stirred vigorously with sterile forceps to clean all the soil from the root surfaces. We avoided collecting any roots that were at the interface of the pot and the soil to avoid unnatural root environments. After removing the cleaned roots, the fluid was centrifuged for 15 min at 10,000 rpm. The supernatant was discarded leaving only the soil fraction behind. This was quickly frozen using liquid nitrogen, and then stored at −80 °C. Bulk soil samples were collected from unplanted pots from ~10 cm below the soil surface. Three biological replicates of each treatment were performed. In total, fifty-four samples were collected.

### DNA Extraction and detection

The genomic DNA for each sample was extracted by Beijing Genomics Institute Tech Solutions Co., Ltd. (Shenzhen, Guangdong, China). DNA concentration and integrality were analyzed by microplate reader and agarose gel electrophoresis.

### Treatment of DNA

All qualified DNA were submitted to BGI Tech Solutions Co., Ltd. (Shenzhen, Guangdong, China) to construct libraries for sequencing. The bioinformatics analysis was carried on with sequencing data (Supplementary method).

## Electronic supplementary material

Supplementary information

Supplementary Table S1

Supplementary Table S2

Supplementary Table S3

Supplementary Table S4

Supplementary Table S5

Supplementary Table S6

Supplementary Table S7

Supplementary Table S8

Supplementary Table S9

Supplementary Table S10

Supplementary Table S11

Supplementary Table S12

Supplementary Table S13

Supplementary Table S14

Supplementary Table S15

Supplementary Table S16
