# Supplementary material for: The Variation in the Rhizosphere Microbiome of Cotton with Soil Type, Genotype and Developmental Stage
Source: Sci Rep. 2017 Jun 21;7:3940. doi: 10.1038/s41598-017-04213-7 (PMC5479781; doi:10.1038/s41598-017-04213-7)
Supplement: Supplementary file 1 — Supplementary information [file 41598_2017_4213_MOESM1_ESM.pdf]

Supplementary information

The Variation in the Rhizosphere Microbiome of Cotton with Soil Type, Genotype and Developmental Stage

Qinghua Qiao, Furong Wang, Jingxia Zhang, Yu Chen, Chuanyun Zhang, Guodong Liu, Hui Zhang, Changle Ma, Jun Zhang

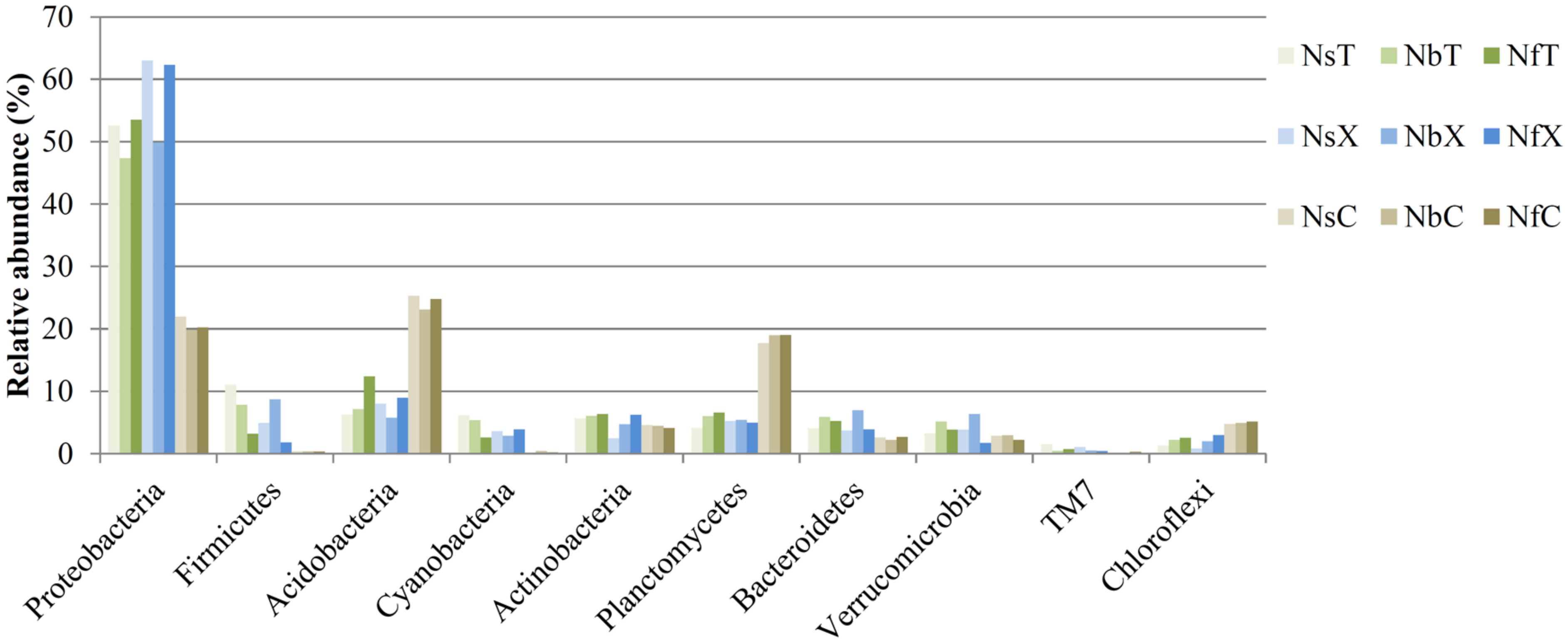

Supplementary Figure 1. Relative abundance of bacterial phyla in nutrient-rich soil.

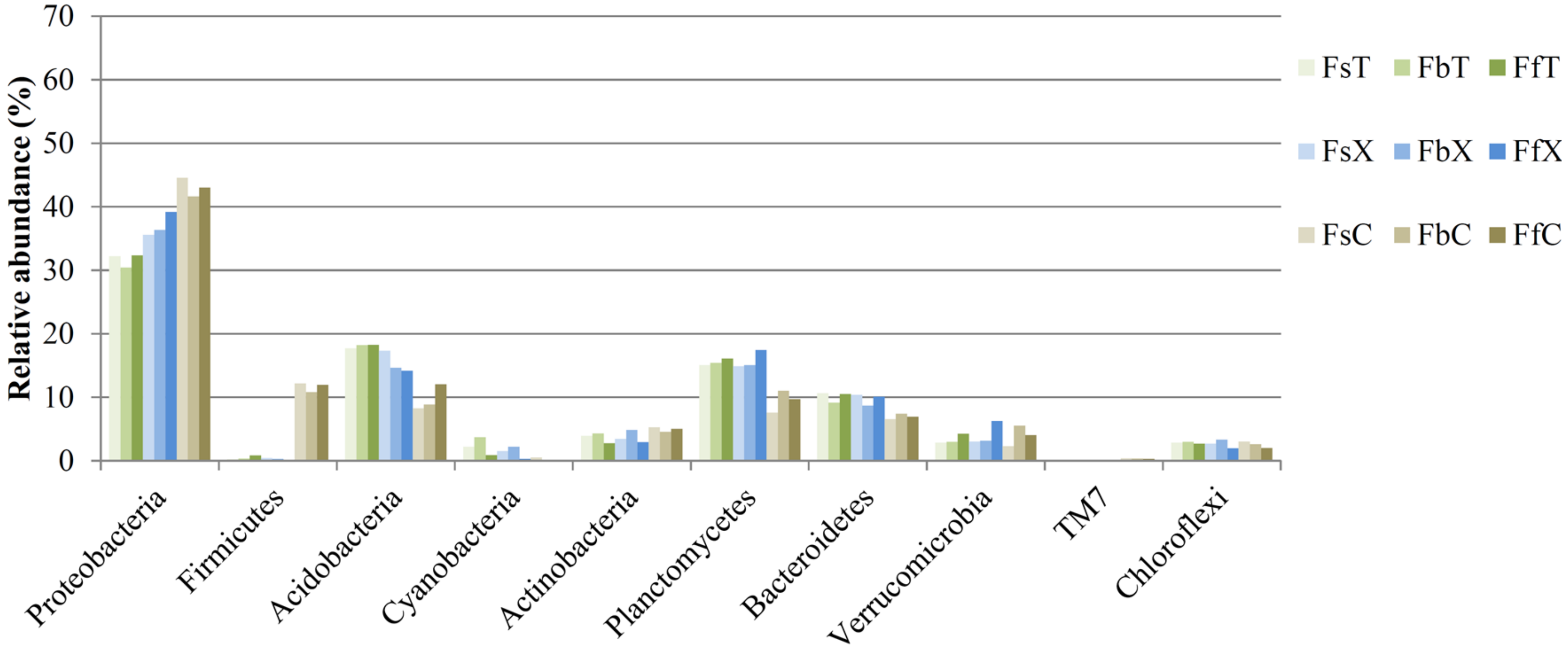

Supplementary Figure 2. Relative abundance of bacterial phyla in continuous cropping field soil.

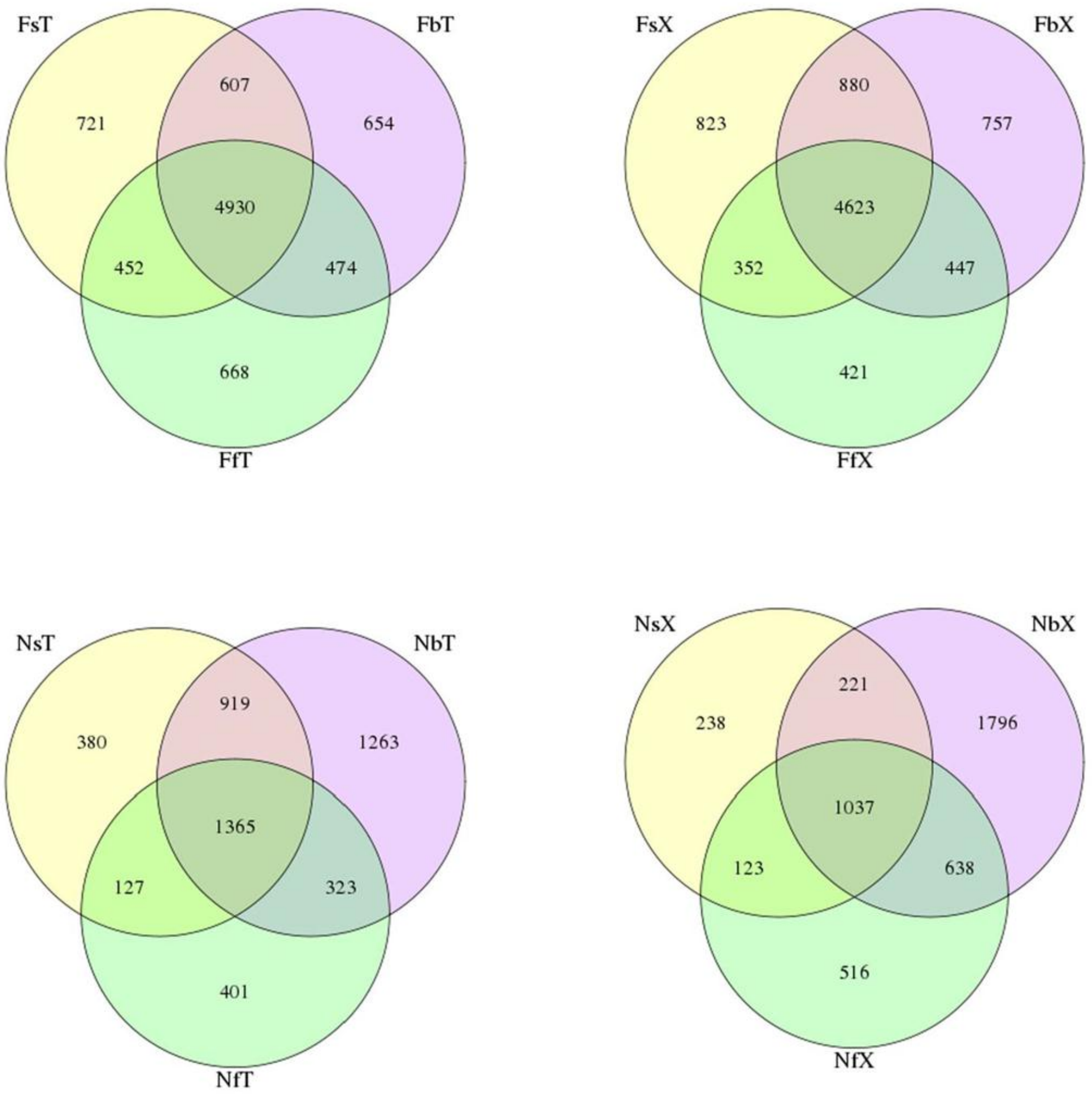

Supplementary Figure 3. OTU numbers of specific and common bacteria under different treatment conditions.

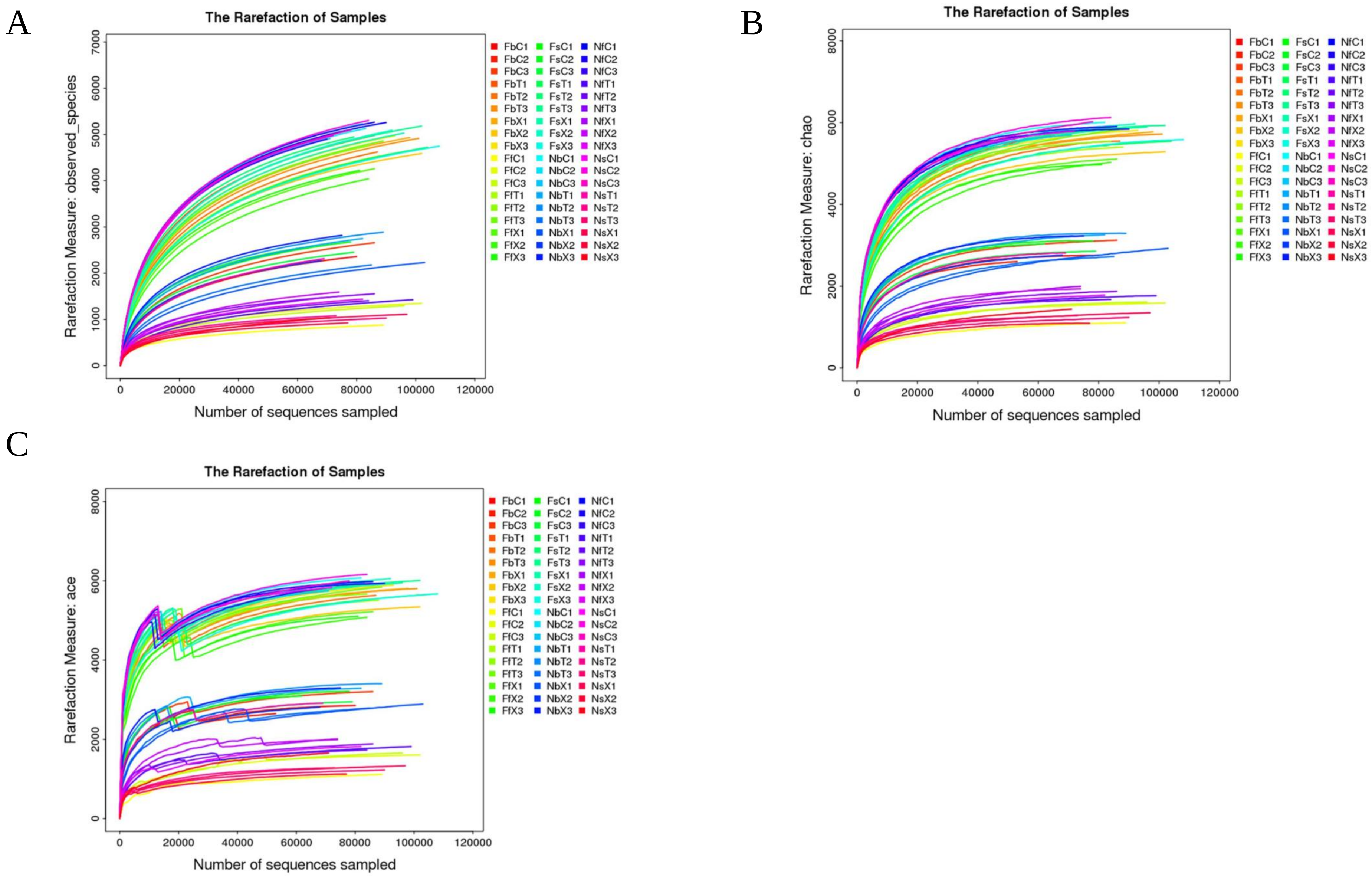

Supplementary Figure 4. Rarefaction of samples.

A: rarefaction of Sobs indices; B: rarefaction of Chao indices; C: rarefaction of ACE indices.

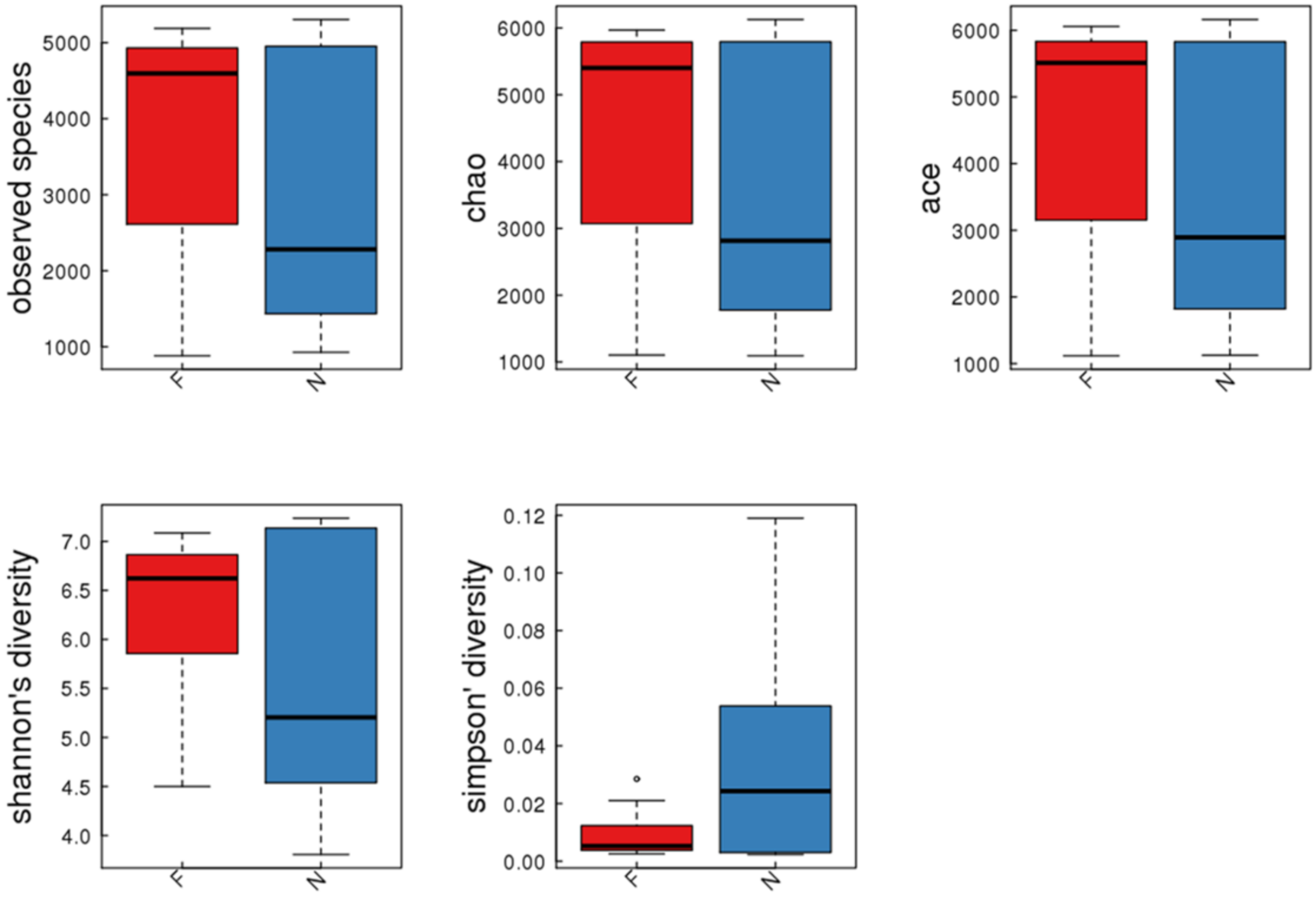

Supplementary Figure 5. α-diversity in different soil type.
From left to right and from top to bottom, box plots are Sobs, Chao, ACE, Shannon, and Simpson indices.

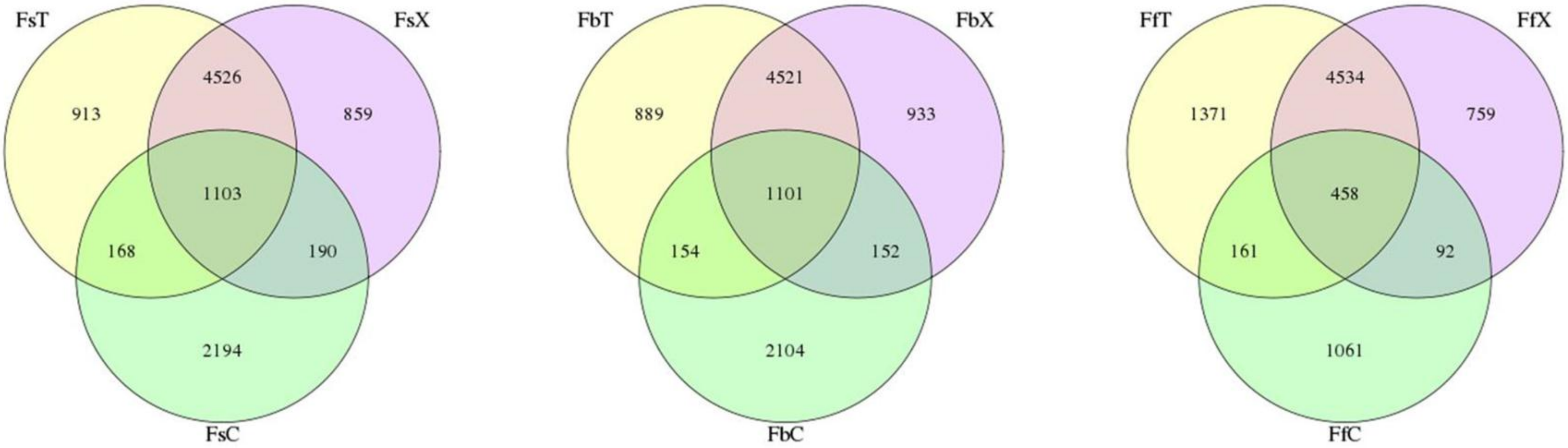

Supplementary Figure 6. OTU numbers of specific and common bacteria between different cultivar rhizosphere and bulk soil at different development stage.

Supplementary Method

Data analysis:

1. To obtain more accurate and reliable results for subsequent bioinformatics analysis, the raw data will be pre-processed to obtain clean data by truncation of sequence reads not having an average quality of 20 bp over a 30 bp sliding window based on the phred algorithm, and trimmed reads having less than 75% of their original length, as well as its paired read, were removed; reads contaminated by the adapter were removed (default parameter: 15 bases overlapped by reads and adapter with a maximum of three bases mismatch allowed); reads with ambiguous bases were removed (N base), along with its paired reads; reads with low complexity were removed (default: reads with 10 consecutive same base). The data processing results are listed in Supplementary Table S1.
2. The high quality paired-end reads were combined to tags when the two paired-end reads overlapped; a consensus sequence was generated by Fast Length Adjustment of Short reads, v1.2.11, and the detailed methods are as follows: 1) Minimal overlapping length: 15 bp; 2) Mismatching ratio of overlapped region: $\leq$ 0.1; paired end reads without overlaps were removed (Supplementary Table S2).
3. The tags were clustered to Operational Taxonomic Units (OTUs) by scripts of USEARCH software (v7.0.1090); the details as follows: 1) The tags were clustered into OTU with a 97% threshold using UPARSE software, and the OTU of unique representative sequences were obtained; 2) Chimeras were filtered out using chimera detection software UCHIME (v4.2.40) by mapping to a gold database (v20110519); 3) All tags were mapped to each OTU representative sequence using USEARCH GLOBAL. Representative OTU sequences were

taxonomically classified using the Ribosomal Database Project Classifier v.2.2 trained on the Greengenes database, and using 0.5 confidence values as a cutoff (Supplementary Table S3).

4. Other analysis:

OTU Venn diagram: Based on the OTU abundance, OTU of each group was listed and a Venn diagram (Fig. 3) was drawn by VennDiagram in the R software package (v3.0.3); then the common and specific OTUs ID were summarized.

Species Annotation: The tags numbering of each taxonomic rank (phylum, class, order, family, genus and species) or OTU in different samples were summarized in a profiling table or histogram, and the histogram was drawn with the software R (v3.0.3) (Fig. 1).

Diversity analysis: Alpha diversity is applied for analyzing the complexity of species diversity for a sample through five indices, including the Sobs, Chao1, ACE, Shannon and Simpson indices. The complexity of each sample is proportional with the first four values, although it has a negative correlation with the Simpson index. Sobs, Chao1 and ACE indices can reflect the species richness of a community, and the rarefaction curve based on these three values could also be used to evaluate if the data produced are sufficient to cover all species in the community. When the curve tends to be smooth, this suggests an adequate amount of data has been produced. Otherwise, when the curve continues to climb with increasing sequencing effort, this shows the samples are very complex, and there are still species that are not uncovered by the sequencing data. The indices were calculated by Mothur software (v1.31.2), and the corresponding rarefaction curves (Supplementary Figure 4) are drawn using R software (v3.0.3). The calculation formula of each index can be found at http://www.mothur.org/wiki/Calculators and the method of drawing rarefaction curve

is as follows, 1) Calculate the number of OTUs based on extracted tags (in multiples of 1000); 2) A rarefraction curve was drawn using the indices calculated with extracted tags. Shannon and Simpson values can reflect the species diversity of the community, and are affected by both species richness and species evenness; that is, the two values also consider the abundance of each species. With the same species richness, when the species evenness is larger, the community diversity is greater.

β diversity analysis was used to evaluate differences in the species complexity of the samples. β diversity analysis was done using QIIME software (v1.80). There are differences in the sequencing depth in different samples, so normalization was introduced: Sequences were extracted randomly according to the minimum sequence number for all samples and the extracted sequences formed a new ‘OTU table biom’ file; next, the β diversity distance could be calculated based on the ‘OTU table biom’ file. Various indices, such as the Bray–Curtis, weighted UniFrac, unweighted UniFrac, and Pearson indices, could be used to measure β diversity, especially the first three values of those listed above. A β diversity heat map was drawn by the 'aheatmap' in package 'NMF' of R software (v3.0.3).

The Unweighted Pair Group Method with Arithmetic mean is a type of hierarchical clustering method using average linkage and was used to interpret the distance matrix produced by β diversity. To measure the robustness of this result to sequencing effort, we performed a jackknifing analysis, wherein 75% of the smallest sample sequences from each sample are chosen at random, and the resulting Unweighted Pair Group Method with Arithmetic mean tree from this subset of data is compared with the tree representing the entire available data set by QIIME (v1.80). This process is repeated with 100 random subsets of data, and the tree nodes which prove more consistent across jackknifed datasets are deemed to be more robust. The

figure is drawn by software R (v3.0.3) (Fig. 6).

The Bray–Curtis distance method and the information entropy method were used to measure the contribution to variation between samples of the different factors; then, we conducted an analysis of variance by the function aov in the R package. Interaction between each two factors were considered. According to the results, for each factor, the contribution rate to the variations of microbial community construction was calculated by the mean square of factor /sum of mean square of all factors.
