# Supplementary material for: The Variation in the Rhizosphere Microbiome of Cotton with Soil Type, Genotype and Developmental Stage
Source: Sci Rep. 2017 Jun 21;7:3940. doi: 10.1038/s41598-017-04213-7 (PMC5479781; doi:10.1038/s41598-017-04213-7)
Supplement: Supplementary file 15 — Supplementary Table S14 [file 41598_2017_4213_MOESM15_ESM.doc]

**Key Laboratory of Plant Nutrition and Fertilizer of Shandong Province**

**Test Report**

No 2014-160-168

| Code Of Sample | |  |  | test items | | | | | | | | | |
| --- | --- | --- | --- | --- | --- | --- | --- | --- | --- | --- | --- | --- | --- |
|  |  | hydrolysable nitrogen mg/kg | available phosphorus mg/kg | available potassium mg/kg | PH | organic material g/kg | exchangeable calcium g/kg | exchangeable magnesium g/kg | exchangeable sodium mg/kg | available cupper mg/kg | available zinc mg/kg | available iron mg/kg | available manganese mg/kg |
| 160 | F1 | 37.10 | 23.20 | 135.0 | 8.34 | 12.64 | 7.50 | 0.69 | 94.0 | 1.66 | 2.74 | 4.08 | 1.62 |
| 161 | F2 | 46.52 | 23.97 | 127.5 | 8.35 | 11.52 | 7.26 | 0.62 | 104.0 | 1.67 | 2.68 | 3.18 | 1.63 |
| 162 | F3 | 39.97 | 24.14 | 130.0 | 8.39 | 12.12 | 7.39 | 0.65 | 99.0 | 1.63 | 2.65 | 4.02 | 1.72 |
| 163 | N1 | 1458.06 | 176.68 | 1685.0 | 4.93 | 45.74 | 2.60 | 0.69 | 185.0 | 2.50 | 4.43 | 1222.46 | 238.51 |
| 164 | N2 | 1521.92 | 206.18 | 1685.0 | 4.77 | 40.62 | 2.43 | 0.68 | 182.0 | 2.81 | 4.45 | 1145.66 | 182.15 |
| 165 | N3 | 1191.99 | 182.24 | 1645.0 | 4.91 | 47.49 | 2.72 | 0.70 | 189.0 | 3.30 | 6.14 | 1245.26 | 247.27 |
| 166 | F+S1 | 35.22 | 19.35 | 84.0 | 8.53 | 10.25 | 6.89 | 0.62 | 87.0 | 1.36 | 2.26 | 2.99 | 1.78 |
| 167 | F+S2 | 36.45 | 20.63 | 85.0 | 8.53 | 10.62 | 6.70 | 0.57 | 89.0 | 1.49 | 2.31 | 3.56 | 1.77 |
| 168 | F+S3 | 35.63 | 20.97 | 82.0 | 8.48 | 10.50 | 6.46 | 0.54 | 89.0 | 1.36 | 2.21 | 3.09 | 1.68 |
|  | 以下空白 |  |  |  |  |  |  |  |  |  |  |  |  |
|  |  |  |  |  |  |  |  |  |  |  |  |  |  |
|  |  |  |  |  |  |  |  |  |  |  |  |  |  |

Notes: F: cotton continuous cropping field soil

N: nutrient-rich soil

F+S: mixture of cotton continuous cropping field soil and sand
